# Chromosome-scale genome assemblies and annotations for Poales species *Carex cristatella*, *Carex scoparia*, *Juncus effusus*, and *Juncus inflexus*

**DOI:** 10.1093/g3journal/jkac211

**Published:** 2022-08-17

**Authors:** Jose Planta, Yu-Ya Liang, Haoyang Xin, Matthew T Chansler, L Alan Prather, Ning Jiang, Jiming Jiang, Kevin L Childs

**Affiliations:** Department of Plant Biology, Michigan State University, East Lansing, MI 48824, USA; National Institute of Molecular Biology and Biotechnology, University of the Philippines, Diliman, Quezon City 1101, Philippines; Department of Plant Biology, Michigan State University, East Lansing, MI 48824, USA; Department of Plant Biology, Michigan State University, East Lansing, MI 48824, USA; Department of Plant Biology, Michigan State University, East Lansing, MI 48824, USA; Department of Plant Biology, Michigan State University, East Lansing, MI 48824, USA; Department of Horticulture, MSU AgBioResearch, Michigan State University, East Lansing, MI 48824, USA; Department of Plant Biology, Michigan State University, East Lansing, MI 48824, USA; Department of Horticulture, MSU AgBioResearch, Michigan State University, East Lansing, MI 48824, USA; Department of Plant Biology, Michigan State University, East Lansing, MI 48824, USA

**Keywords:** *Carex cristatella*, *Carex scoparia*, *Juncus effusus*, *Juncus inflexus*

## Abstract

The majority of sequenced genomes in the monocots are from species belonging to Poaceae, which include many commercially important crops. Here, we expand the number of sequenced genomes from the monocots to include the genomes of 4 related cyperids: *Carex cristatella* and *Carex scoparia* from Cyperaceae and *Juncus effusus* and *Juncus inflexus* from Juncaceae. The high-quality, chromosome-scale genome sequences from these 4 cyperids were assembled by combining whole-genome shotgun sequencing of Nanopore long reads, Illumina short reads, and Hi-C sequencing data. Some members of the Cyperaceae and Juncaceae are known to possess holocentric chromosomes. We examined the repeat landscapes in our sequenced genomes to search for potential repeats associated with centromeres. Several large satellite repeat families, comprising 3.2–9.5% of our sequenced genomes, showed dispersed distribution of large satellite repeat clusters across all *Carex* chromosomes, with few instances of these repeats clustering in the same chromosomal regions. In contrast, most large *Juncus* satellite repeats were clustered in a single location on each chromosome, with sporadic instances of large satellite repeats throughout the *Juncus* genomes. Recognizable transposable elements account for about 20% of each of the 4 genome assemblies, with the *Carex* genomes containing more DNA transposons than retrotransposons while the converse is true for the *Juncus* genomes. These genome sequences and annotations will facilitate better comparative analysis within monocots.

## Introduction

More than 140 plant genome sequences have been published for species in the monocots, but within this large plant clade, the number of species with sequenced genomes is heavily skewed toward members of the economically important Poaceae (https://www.plabipd.de/plant_genomes_pa.ep). More than 80 genomes have been published from species in the Poaceae, a member of Poales, an order with 14–16 families (Bremer 2002; Bouchenak-Khelladi *et al.* 2014; http://www.mobot.org/MOBOT/research/APweb/). Only 5 genomes have been sequenced from other species within Poales: 3 from Bromeliaceae, 1 from Typhaceae, and 3 from Cyperaceae (Redwan *et al.* 2016; Chen *et al.* 2019; Can *et al.* 2020; Liu *et al.* 2021; Qu *et al.* 2022; Widanagama *et al.* 2022). Cyperaceae and Juncaceae are 2 sister families within Poales with about 5,000 and 440 species, respectively (Goetghebeur 1998; Roalson 2005; Larridon *et al.* 2021). New high-quality genomes from these families will help to strengthen support for the phylogeny of Poales (Bouchenak-Khelladi *et al.* 2014).

In addition to their position within Poales, members of Cyperaceae and Juncaceae are also of interest for having species with holocentric chromosomes (Hipp 2007; Håkansson 2010; Burchardt *et al.* 2020). While holocentrism has been reported in *Carex* and *Rhynchospora* in Cyperaceae (Heilborn 2010; Marques *et al.* 2015; Escudero *et al.* 2016; Ribeiro *et al.* 2017), only members of *Luzula* in Juncaceae are confirmed as having holocentric chromosomes (Haizel *et al.* 2005; Heckmann *et al.* 2013). Four species in *Juncus* have recently been reported to have monocentric chromosomes (Guerra *et al.* 2019), but that study has been questioned due to the method used to classify those chromosomes as monocentric (Krátká *et al.* 2021). Centromeres are defined by the chromosomal positions where CenH3 histones are incorporated into the local nucleosomes, and centromeric regions often contain long arrays of repetitive DNA sequences, mostly satellite repeats and transposons (Henikoff *et al.* 2001; Jiang *et al.* 2003). While the satellite DNA sequences are not a defining feature of centromeres, they are often found to be associated with centromeres, which is likely associated with the fact that satellite repeats are potentially favorable to confer translational and rotational phasing on CenH3 nucleosomes (Hasson *et al.* 2013; Zhang *et al.* 2013; Yang *et al.* 2018).

With the goal of allowing better comparative analyses within monocots and increasing the diversity of sequenced genomes within Poales, we report here on the sequencing and annotation of the genomes of *Carex cristatella* and *Carex scoparia* from Cyperaceae and *Juncus effusus* and *Juncus inflexus* from Juncaceae. *Carex* is a large genus with a world-wide distribution and more than 2,000 members (Reznicek 1990). *Carex cristatella* and *C. scoparia* are native to North American wetland meadows (Gleason and Cronquist 1991; Kruse and Groninger 2003). *Juncus* is a moderately sized genus with more than 300 species (Kirschner *et al.* 1999). *Juncus effusus* and *J. inflexus* are wetland species with wide distributions. *Juncus effusus* is native to North America, South America, Europe, Asia, and Africa (Kirschner 2002), and *J. inflexus* is native to Africa, Asia, and Europe (Richards and Clapham 1941). These 4 high-quality genome assemblies will aid researchers in comparative genomic analyses within Poales and will allow an examination of their satellite DNA content, which may provide insight into centromere organization in these species.

## Materials and methods

### Plant material

Seeds for *C. cristatella* and *C. scoparia* were obtained from Roundstone Native Seed (Upton, KY). *Juncus effusus* plants were purchased from Perennial Farm Marketplace (Glen Arm, MD), and *J. inflexus* plants were obtained from Jelitto Staudensamen GmbH (Schearmstedt, Germany). Voucher specimens for all 4 species have been deposited at the Michigan State University Herbarium (Supplementary Table 1).

### Plant growth and tissue collection

Cold-stratified *C. cristatella* and *C. scoparia* seeds were germinated in Greens Grade soil (Profile Products LLC, IL, USA) under a 16-h photoperiod with day/night temperatures of 24°C/18°C. *Juncus effusus* and *J. inflexus* plants were grown in a peat soil mix in a greenhouse under natural light with daily temperatures kept to a maximum of 28°C. Two-month-old *C. cristatella* and *C. scoparia* plants were kept in the dark for 4 days prior to DNA extraction, and 2-month-old green stems from *J. effusus* and *J. inflexus* were used for DNA extraction. For RNA extraction, 2-month-old plant tissues including roots and crowns from all 4 species, leaves and culm from *C. cristatella* and *C. scoparia* and green stems from *J. effusus* and *J. inflexus* were collected, frozen in liquid nitrogen, and stored at −80°C before processing. Additional samples for RNA extraction were obtained from the leaves of 2-month-old *C. cristatella* and *C. scoparia* plants subjected to drought stress for 2 weeks by withholding watering. *Juncus effusus* and *J. inflexus* plants were also subjected to 2 weeks of drought stress, and stems were collected for RNA extraction.

### DNA and RNA isolation

High-molecular-weight DNA from *Carex* leaf and *Juncus* stem tissues was extracted using the Genomic-tip 20/G kit (Qiagen, Hilden, Germany). Frozen tissues were ground to a fine powder with mortar and pestle with liquid nitrogen. Ground tissues were resuspended in lysis buffer G2 containing 0.5 mg/mL Driselase (Cat. No. D8037, Sigma-Aldrich, MO, USA) and 0.5 mg/mL Lysing Enzymes (Cat. No. L1412, Sigma-Aldrich, MO, USA) and incubated at 37°C for an h with gentle agitation. The tissue lysates were then supplemented with 20 µg/mL DNase- and protease-free RNase A (Thermo Fisher Scientific, MA, USA) and incubated for an additional hour at 37°C. For the final enzyme pretreatment, the lysates were further supplemented with 0.8 mg/mL Proteinase K (Qiagen, Germany) and incubated for 2 h at 50°C with gentle agitation. Insoluble debris was pelleted out by centrifuging the lysate for 20 min at 15,000 × g, and the clarified lysate was passed through a Buffer QBT-equilibrated Genomic-tip column. The column was washed 4 times with Buffer QC, and DNA was eluted with Buffer QF. To pellet the DNA, 0.7 volumes of room-temperature isopropanol were added to the eluate and centrifuged for 20 min at 15,000 × g, followed by a final wash of the DNA pellet with ice-cold 70% ethanol. Precipitated DNA was resuspended in Tris EDTA buffer by an overnight incubation at room temperature, and DNA cleanup was performed with an Amicon Ultra-2 centrifugal filter with a 100,000 MWCO (Millipore, MA, USA). Prior to long-read sequencing with Nanopore (Oxford Nanopore Technologies Ltd, New York, USA), input DNAs were processed with the Standard Short Read Eliminator Kit (Circulomics Inc., MD, USA) to reduce the number of short reads less than 25 kb.

For RNA isolation, tissues were ground in liquid nitrogen and processed with an RNeasy Plant Mini Kit (Qiagen, Germany). All RNA samples were treated with Turbo DNA-free DNase (Thermo Fisher Scientific, USA). Culm, crown, root, and leaf tissues, either from well-watered or water-stressed plants, of *C. cristatella* and *C. scoparia* were used for RNA extraction. Root, crown, and green stem tissues from well-watered and water-stressed plants of *J. effusus* and *J. inflexus* were also used for RNA extraction.

### DNA and RNA sequencing

DNA libraries for long-read sequencing with Nanopore (Oxford Nanopore Technologies Ltd, USA) were prepared with 1–5 µg DNA and the standard ligation sequencing kit SQK-LSK109 (Oxford Nanopore Technologies Ltd, USA). Assessment of the prepared libraries was performed with the Agilent 4200 TapeStation (Agilent Technologies, CA, USA), and libraries were loaded either on the FLO-MIN106 or FLO-MIN106D flow cells for sequencing on the GridION X5 platform (Oxford Nanopore Technologies Ltd, USA). Base calling in real-time was performed by Guppy ver. 3.0.6 or ver. 3.2.6 using the High Accuracy Model (https://github.com/nanoporetech).

One microgram of extracted genomic DNA was also prepared for short-read sequencing. DNA libraries for *C. cristatella* and *C. scoparia* were created with an Illumina TruSeq Nano DNA Library Preparation Kit (Illumina Inc., CA, USA). DNA libraries for *J. effusus* and *J. inflexus* were prepared with a Celero DNA-Seq Library Preparation Kit (Tecan Genomics, Inc., CA, USA). Sequencing was performed with the paired-end 150-bp reads on Illumina HiSeq 4000 or NextSeq 500 instruments. Quality trimming and adapter clipping of all raw Illumina sequencing reads were done using Trimmomatic ver. 0.36 with default parameters for paired-end reads (Bolger *et al.* 2014).

Long-read Nanopore RNA-sequencing libraries were prepared using the SQK-PCS109 PCR-cDNA Sequencing Kit and SQK-PBK004 PCR Barcoding Kit (Oxford Nanopore Technologies Ltd, USA). Total RNA from tissues described above from *C. cristatella*, *C. scoparia*, *J. effusus*, and *J. inflexus* were individually barcoded. Pooled RNA samples from each species were loaded into individual FLO-MIN106D flow cells for sequencing on a GridION instrument, and base calling was performed in real-time by Guppy ver. 3.2.10. Adapters were clipped from the Nanopore raw reads using Porechop ver. 0.2.4 (https://github.com/rrwick/Porechop) and subsequent reads with lengths less than 300 bases were removed with NanoFilt ver. 2.5.0 (De Coster *et al.* 2018).

### Genome size estimation

The sizes of the genomes from the *Carex* and *Juncus* species were estimated by flow cytometry and k-mer analysis. *C*-values for the 2 *Carex* and 2 *Juncus* species were estimated using fresh leaves for flow cytometry analysis at the Flow Cytometry Core Laboratory at the Benaroya Research Institute. Leaf samples from *Oryza sativa* and *Arabidopsis thaliana* were assayed by flow cytometry at the same time and used as standards. In addition, Jellyfish was used to estimate genome sizes and heterozygosity using k-mer analysis with 31-mers from the trimmed and filtered reads from Illumina DNA libraries (Marçais and Kingsford 2011), and k-mer profiles were visualized with GenomeScope (Vurture *et al.* 2017).

### Chromosome preparation and counting

Root tips were harvested from greenhouse-grown plants and pretreated with nitrous oxide at a pressure of 160 psi (∼10.9 atm) for 30 min. The root tips were then treated with fixative solution (3 ethanol: 1 acetic acid) and kept at 22°C until enzyme treatment. An enzymatic solution with 4% cellulase (Yakult Pharmaceutical, Tokyo, Japan), 2% pectinase (Plant Media, Dublin, OH, USA), and 2% pectolyase (Sigma Chemical, St. Louis, MO, USA) was used to digest the root tips for 1 h at 37°C. Chromosomes were prepared using a stirring method (Xin *et al.* 2020) and were counterstained with 4',6-diamidino-2-phenylindole (DAPI) in VectaShield antifade solution (Vector Laboratories, Burlingame, CA, USA). Images were captured using a QImaging Retiga EXi Fast 1394 CCD camera (Teledyne Photometrics, Tuscon, AZ, USA) attached to an Olympus BX51 epifluorescence microscope. Images were processed with Meta Imaging Series 7.5 software. The final contrast of the images was processed using Adobe Photoshop software (Adobe, San Jose, CA, USA). Chromosome counting was carried out in at least 20 metaphase spreads per species.

### Genome assembly, error correction, polishing, and quality assessment

Raw Nanopore DNA reads with mean Q-scores greater than 7 were used and processed with a pipeline consisting of NanoFilt ver. 2.5.0 (De Coster *et al.* 2018) to filter reads less than 1,000 nucleotides, Porechop ver. 0.2.4 (https://github.com/rrwick/Porechop) to trim for adapter sequences and Filtlong ver. 0.2.0 (https://github.com/rrwick/Filtlong) to filter out the worst 10% of reads based on read quality (Supplementary Table 2). Sequences were then assembled with the genome assembler Flye ver. 2.6 (Kolmogorov *et al.* 2019) with 2 rounds of polishing (“-i 2”) and minimum overlap between reads of either 5,000 for *C. scoparia*, *J. effusus*, and *J. inflexus* or 1,000 for *C. cristatella* (“-m 5000” or “-m 1000”). Haplotype duplicates from the initial Flye assemblies were removed using purge_dups ver. 1.0.1 (Guan *et al.* 2020).

The draft assemblies were polished for 4 rounds using the NanoFilt- and Porechop-processed raw reads with Racon ver. 1.4.0 (Vaser *et al.* 2017) and one round with Medaka ver. 0.10.0 (https://github.com/nanoporetech/medaka) with the r941_min_high mode. Bwa-mem (bwa ver. 0.7.15) was then used to align Illumina paired-end reads to the draft genome assemblies (Li 2013), and the resulting alignment files were used for one round of polishing with Pilon ver. 1.23 (Walker *et al.* 2014).

### Hi-C library preparation and scaffolding

The Proximo Plant Hi-C Kit (Phase Genomics, Inc., WA, USA) was used to prepare Hi-C libraries from 1 to 1.5 g young and fresh *Carex* leaves and *Juncus* green stems following the manufacturer’s recommendations. Libraries were sequenced to obtain 150-nt paired-end reads on an Illumina HiSeq 4000. Scaffolding of the assembled contigs was processed using the 3D-DNA pipeline (Dudchenko *et al.* 2017). Hi-C reads were mapped into the draft assembly with Juicer ver. 1.6 using the default parameters in bwa ver. 0.7.15 (Li 2013; Durand, Shamim *et al.* 2016), and contact maps generated by the “run-asm-pipeline.sh” script of 3D-DNA ver. 180922 were manually reviewed and corrected in Juicebox ver. 1.11.08 (Durand, Robinson *et al.* 2016). Final genome assemblies were obtained by processing the modified contact maps with the “run-asm-pipeline-post-review.sh” script of 3D-DNA ver. 180922 using the parameters “-g 300 –sort-output.” Genome assembly statistics were calculated using QUAST ver. 5.0.2 (Mikheenko *et al.* 2018), and gene space completeness of the genome assemblies was assessed using BUSCO ver. 3.1.0 using both the Embryophyta_db10 and Poales_db10 gene databases (Waterhouse *et al.* 2018). Illumina paired-end reads were remapped to the final manually reviewed Hi-C scaffolds using bwa-mem for the calculation of correct read pair mapping percentages.

### Genome annotation

Before gene annotation, repetitive sequences were identified in order to mask the genome. Repetitive sequences were first identified using RepeatModeler (http://www.repeatmasker.org/RepeatModeler/). The repeat library output by RepeatModeler contains both known and unknown repeats. Long terminal repeat (LTR) retrotransposons were collected using LTR_retriever (Ou and Jiang 2018). The repeat libraries generated by RepeatModeler and LTR_retriever were combined to mask the genomic DNA using RepeatMasker (http://www.repeatmasker.org/).

Genes were annotated using MAKER2 (Holt and Yandell 2011). Plant protein sequences were downloaded from SwissProt, and predicted proteins were obtained from the Rice Genome Annotation Project (version 7; Ouyang *et al.* 2007; Kawahara *et al.* 2013; Boutet *et al.* 2016). These protein sequences were aligned to the scaffolded genome assemblies using Exonerate with the parameters “–model protein2genome –bestn 5 –minintron 10 –maxintron 3000” (Slater and Birney 2005). Protein alignments in GFF format were then used as protein evidence within MAKER2. Filtered Nanopore cDNA reads were mapped to the scaffolded genome assemblies with minimap2 ver. 2.17 (Li 2018) using the parameters “-N 1 -ax splice -g2000 -G5k,” and the transcript sequences were assembled with StringTie2 ver. 2.0 (Kovaka *et al.* 2019) using the parameters “-m 300 -t -c 2.5 -f 0.05 -g 250.” The assembled transcript alignments from StringTie2 in GFF format were used as transcript evidence within MAKER2. Initial gene models were created by MAKER2 using the est2genome parameter.

Predicted genes from the first round of MAKER2 were used to train the SNAP (Korf 2004) and Augustus (Stanke *et al.* 2008) gene prediction programs, which were then used for final gene prediction with a second round of MAKER2. For each genome, a MAKER standard gene set was created that consisted of gene predictions that had evidence support or that contained a Pfam domain. TE-related predictions from the MAKER standard gene set were identified and removed following the recommendations of Bowman *et al.* (2017), and fused gene models in the remaining gene set were corrected using deFusion with manual review (https://github.com/wjidea/defusion). Predicted genes with CDSes less than 150 nucleotides long were removed. Protein domains and motifs were annotated using InterProScan (Jones *et al.* 2014), and gene functions were assigned according to the best match to annotated rice proteins using BLAST+ ver. 2.9.0 with parameters “-evalue 1e-6 -max_hsps 1 -max_target_seqs 5” (Camacho *et al.* 2009).

### Gene family analysis

Predicted protein sequences from 4 plant species with whole-genome sequences were used for gene family clustering analysis with the *Carex* and *Juncus* predicted proteins. The *O. sativa* protein sequences were downloaded from the Rice Genome Annotation Project (Ouyang *et al.* 2007; Kawahara *et al.* 2013). *Ananas comosus* and *Sorghum bicolor* protein sequences were obtained from Phytozome (https://phytozome-next.jgi.doe.gov/), and *Musa balbisiana* protein sequences were downloaded from the Banana Genome Hub (https://banana-genome-hub.southgreen.fr/). OrthoFinder ver 2.5.2 was used to cluster similar genes and identify orthogroups (Emms and Kelly 2019). Subsets of *Carex*-specific, *Juncus*-specific, and Poaceae-specific gene families were extracted for gene ontology (GO) enrichment analysis following the procedure of Bonnot *et al.* (2019), and plots were generated using CirGO (Kuznetsova *et al.* 2019).

### Phylogenetic tree construction

A phylogenetic tree was constructed with the predicted proteins from the newly annotated *Carex* and *Juncus* species and predicted proteins from an additional 7 Poales species (*A. comosus, Carex littledalei, M. balbisiana, O. sativa, Puya raimondii, S. bicolor, Typha latifolia*) and *Asparagus setaceus* as an outgroup (Kawahara *et al.* 2013; Ming *et al.* 2015; McCormick *et al.* 2018; Wang *et al.* 2019; Can *et al.* 2020; Li *et al.* 2020; Liu *et al.* 2021; Widanagama *et al.* 2022). Single-copy gene orthogroups containing genes from all 12 species were identified after OrthoFinder analysis, and a phylogenetic tree was constructed with RAxML ver. 8.2.12 (Stamatakis 2014). Phylogeny was inferred using RAxML with the PROTGAMMAJTTDCMUT model and 1,000 bootstrap replicates. Finally, an MCMCTree from 459 single-copy gene clusters from all 12 species was constructed with PAML v4.8a using the global clock model (Yang 2007). Calibration of the tree used the divergence times between *A. setaceus* and *M. balbisiana* (108.4–122 Mya), *O. sativa* and *S. bicolor* (40.3–51.9 Mya), *T. latifolia* and *A. comosus* (95.6–112.3 Mya), and *P. raimondii* and *O. sativa* (91.1–114.0 Mya) (Hedges *et al.* 2015; Kumar *et al.* 2017). The program nwkit mcmctree (https://github.com/kfuku52/nwkit/wiki/nwkit-mcmctree) was used to prepare the input tree file for mcmctree in PAML (Yang 2007). The phylogenetic tree with divergence times was visualized by FigTree v1.4.4 (http://tree.bio.ed.ac.uk/software/figtree/).

### tRNA annotation and analysis

High-confidence tRNA genes were identified by tRNAscan-SE ver. 2.0.5 (Chan *et al.* 2021) and Infernal ver. 1.1.2 (Nawrocki and Eddy 2013), with unique tRNA sequences identified following the analysis of Tang *et al.* (2009). Genomes used for comparative tRNA analyses with the *Carex* and *Juncus* genomes included those from *A. comosus*, *M. balbisiana, O. sativa*, and *S. bicolor*. tRNA gene total copy number correlation between *O. sativa* and the other 7 species was examined by Spearman's rank correlation test using the cor.test() function in RStudio ver. 3.6.2.

To determine the correlation between genome size and the number of tRNA genes, 15 additional monocot genome sequences were downloaded from GenBank (Benson *et al.* 2013) and Phytozome (Goodstein *et al.* 2012). Genome sequences downloaded from GenBank included *Dactylis glomerata, Carex littledalei*, and *Phoenix dactylifera* while sequences obtained from Phytozome included *Asparagus officinalis, Brachypodium distachyon, Dioscorea alata, Hordeum vulgare, Miscanthus sinensis, Musa acuminata, Panicum hallii, Setaria viridis, Spirodela polyrhiza, Triticum aestivum, Zea mays*, and *Zostera marina*. tRNAscan-SE ver. 2.0.5 (Chan *et al.* 2021) was then used to identify the number of high-confidence tRNA genes from each of these genomes.

### Satellite repeat identification

Satellite repeats were identified in the genome assemblies of *C. cristatella*, *C. scoparia, J. effusus*, and *J. inflexus* using Tandem Repeat Finder (TRF, version v4.09.1) following the approaches outlined in Melters *et al.* (2013) and VanBuren *et al.* (2015). For each species, repeats were sorted by period size, and total copy numbers for each period size were determined. Base sequences from repeats with highly represented period sizes were self-concatenated to form triples and used to create a BLAST database, and the unconcatenated repeats were aligned to the BLAST database (BLAST+ version 2.2.30) (Camacho *et al.* 2009). Groups of TRF-identified repeats that had BLAST similarities to each other using a BLAST *e*-value cutoff of 1e−20 were identified. GFF files for the repeat groups with the largest numbers of repeat instances were created, sorted, and merged with the BEDTools merge function (version 2.23.0) because TRF often identifies repeat variants from the same genomic region (Quinlan and Hall 2010). The total number of bases covered by the repeats in each group was calculated from the merged repeat GFF files. For each species, the 3 largest repeat groups as determined by total bases were identified and used to manually create consensus sequences of satellite repeats.

## Results and discussion

### Genome size estimates

Genome size estimates for *C. cristatella*, *C. scoparia*, *J. effusus*, and *J. inflexus* were calculated by both flow cytometry and k-mer analysis. All 4 species have modest genome sizes between 196 and 317 Mbp as determined by these methods (Table 1). The flow cytometry estimates tended to be smaller than k-mer-based estimates. The k-mer analyses with a k-value of 31 indicated that all 4 species are diploids with very low heterozygosity between 0.25% and 1.25% (Supplementary Fig. 1).

**Table 1.** Estimates of genome sizes for *Carex* and *Juncus* by flow cytometry and k-mer statistics.

Genome size (Mb)	*C. cristatella*	*C. scoparia*	*J. effusus*	*J. inflexus*
Flow cytometry	255.2 ± 13.1	268.8 ± 9.7	198.9 ± 4.6	286.4 ± 4.7
k-mer statistics				
31-kmer	317.3	294.5	225.5	196.0

### Genome assembly

High-quality chromosome-scale genome assemblies were developed for all 4 species. Initial Oxford Nanopore long-read genome assemblies for these species were highly contiguous with 206–984 contigs each (Table 2). Scaffolding with Hi-C sequencing produced 35 chromosome-scale scaffolds for *C. cristatella* (Supplementary Fig. 2). This was consistent with the haploid chromosome number observed by karyotyping (*n* = 35, 2*n* = 70, Supplementary Fig. 3) and with published karyotype analysis for *C. cristatella* (Löve 1981; Hipp 2007; Chung *et al.* 2011; Escudero *et al.* 2013). The Hi-C scaffolded assembly for *C. scoparia* has 33 scaffolds, but karyotype analysis indicates that our *C. scoparia* has a haploid chromosome number of 31 (*n* = 31, 2*n* = 62, Supplementary Figs. 4 and 5). Chromosome numbers in *C. scoparia* are variable with reports ranging between 28 and 35 (Löve 1981; Hipp 2007). The observed number of chromosomes in our example of *C. scoparia* indicates that while most scaffolds in the assembly are chromosome-scale, 3 or 4 scaffolds likely represent partial chromosomes. Published chromosome counts have reported haploid chromosome numbers of 20 and 21 for *J. effusus* and 19 and 20 for *J. inflexus* (Löve and Löve 1944; Snogerup 1963; Löve 1981). There were 21 Hi-C-based chromosome-sized scaffolds in our genome assembly for *J. effusus* greater than 7.5 Mbp in length, but an additional 340 scaffolds greater than 5 kbp and totaling 1.2 Mbp in size could not be incorporated into the largest scaffolds (Supplementary Figs. 6 and 7). Karyotyping also shows *J. effusus* to have a haploid chromosome number of 21 (2*n* = 42, Supplementary Fig. 8). The *J. inflexus* Hi-C scaffolded assembly has 21 chromosome-sized scaffolds (Supplementary Fig. 9), which is in agreement with karyotyping analysis which showed a haploid chromosome number of 21 (2*n* = 42, Supplementary Fig. 10).

**Table 2.** Assembly statistics of the Hi-C-scaffolded genome assemblies of *Carex* and *Juncus*.

	*C. cristatella*	*C. scoparia*	*J. effusus*	*J. inflexus*
**No. of contigs (≥5 kbp)^*a*^**	206	984	304	223
**No. of scaffolds (≥5 kbp)**	35	33	361	21
**No. of scaffolds (≥50 kbp)**	35	33	76	21
**No. of scaffolds (≥1 Mbp)**	35	33	23	21
**Total length**	301,638,393	298,037,631	224,468,077	267,557,116
**GC (%)**	33.86	33.76	32.64	33.18
**No. of *N*’s per 100 kbp**	18.60	70.96	140.60	40.25
**LAI**	10.20	11.13	7.94	7.15

a Assembly statistics before scaffolding.

Additional analyses indicate that the final genome assemblies for *C. cristatella*, *C. scoparia*, *J. effusus*, and *J. inflexus* are nearly complete. The sizes of the final genome assemblies for these 4 species ranged from 224 to 302 Mbp and were toward the higher genome size estimates calculated by flow cytometry and k-mer analysis (Tables 1 and 2). Illumina reads were mapped to the final genome assemblies at rates greater than 97% indicating a very high degree of completeness for all 4 genomes (Supplementary Table 3). The assemblies of all 4 species were also analyzed with BUSCO to determine gene space completeness using the BUSCO Embryophyta gene database. The BUSCO results indicate that all 4 genomes are well assembled as all species had very high complete gene scores (95.4–96.4%) and very low duplication rates (2.2–3.2%) (Table 3). The number of missing BUSCO genes in the 4 assemblies was also very low (2.6–3.2%). The 4 assemblies were also analyzed with the BUSCO Poales gene database, which was created using 11 Poaceae species and one Bromeliaceae species (Waterhouse *et al.* 2018). The results with the Poales database were very poor with complete genes between 72.5% and 74.4% and missing genes between 22.9% and 24.6% (Supplementary Table 4). The difference in the results using these 2 BUSCO databases suggests that the reliance on only Poaceae and Bromeliaceae species in the Poales gene database does not truly represent Poales conserved single-copy genes.

**Table 3.** Gene space completeness of the genome assemblies of *Carex* and *Juncus* as determined by BUSCO using the Embryophyta_db10 database (*n* = 1,375).

Classification	*C. cristatella* (%)	*C. scoparia* (%)	*J. effusus* (%)	*J. inflexus* (%)
Complete (C)	96.1	96.4	95.4	95.9
Complete and single-copy (S)	92.8	93.1	93.2	93.6
Complete and duplicated (D)	3.3	3.3	2.2	2.3
Fragmented (F)	0.7	0.6	2.0	1.5
Missing (M)	3.2	3.0	2.6	2.6

LTR assembly index (LAI) values were calculated for each assembly and were found to be moderate to low values (Table 2). While these LAI values have been characterized as representing reference or draft-grade genome assemblies, LAI scores are not only positively correlated with genome completeness, but they are also negatively correlated with percentage of LTR content in the genome (Ou *et al.* 2018). The 4 species contain 6–16% of LTR elements, which is low compared to that in most plant genomes. All 4 of these species have relatively high amounts of tandem repeats, ranging from 3.2% to 9.5% (Table 4 and discussion below). Tandem repeats may interfere with the identification of intact LTR elements (Ou and Jiang 2018), thus resulting in apparently low LAI scores. Moreover, LAI scores have also been found to be lower for small genomes. The small sizes of these genomes, low percentage of LTR element content and high abundance of tandem repeats may be partly responsible for the low LAI scores. Other measures of assembly quality (e.g. assembly contiguity, BUSCO analysis and read remapping) indicate that these genome assemblies are excellent chromosome-scale assemblies, and so, the commonly used LAI metric for genome assembly completeness may not be a good measure for these small *Carex* and *Juncus* genomes with their high percentages of tandem repeats.

**Table 4.** Fraction of repetitive sequences in the *Carex* and *Juncus* genomes involved in this study.

Class	Subclass	Superfamily	Genome fraction (%)			
			*C. cristatella*	*C. scoparia*	*J. effusus*	*J. inflexus*
Class I	LTR	LTR/*Copia*	4.275	3.677	10.394	10.387
		LTR/*Gypsy*	2.426	2.276	4.388	5.044
		LTR/Other	0.158	0.370	0.169	0.190
	Non-LTR	LINE	1.367	1.238	1.984	1.938
		SINE	ND	0.003	0.011	0.007
	Total Class I		8.226	7.563	16.946	17.566
Class II	TIR	CACTA	1.574	4.054	0.527	0.865
		*hAT*	2.994	3.992	1.323	1.528
		MULE	3.019	2.815	1.559	2.394
		*PIF/Harbinger*	0.123	0.206	0.085	0.121
		*Tc1/Mariner*	0.018	0.037	ND	0.181
	Non-TIR	*Helitron*	0.457	0.522	0.307	0.277
	Other		1.788	0.052	0.016	0.023
	Total Class II		9.973	11.679	3.817	5.409
Total TEs			18.199	19.242	20.763	22.975
Satellite Repeats^*a*^			3.218	4.003	9.540	4.126
Other Repeats^*b*^			18.625	16.685	15.333	15.633
Total Repeats			40.042	39.930	48.636	42.734

a Satellite repeats identified by TRF and designated as CR1, CR2, CR3, JR1, JR2, and JR3.

b Other repeats include simple repeats, rRNA, tRNA, snRNA, and other unclassified repeats.

ND, not detected.

### Transposable element analysis

For the *C. cristatella*, *C. scoparia*, *J. effusus*, and *J. inflexus* genomes, recognizable TEs account for about 20% (ranging from 18% to 23%) of the genome assemblies (Table 4). This is likely an underestimate since a large portion of the unclassified repeats could also be TEs. Although transposable elements (TEs) in these *Carex* and *Juncus* genomes are not as abundant as that for plants with larger genomes such as *Zea mays* (Schnable *et al.* 2009; Ou and Jiang 2018), the composition of TEs in these 4 species demonstrates a variety of unique features. First of all, the *C. cristatella* and *C. scoparia* genomes contain a higher fraction of DNA transposons than retrotransposons, and this is unusual because most plant genomes contain higher fractions of retrotransposons (class I) than DNA transposons (class II) (Supplemental Fig. 11) (Kejnovsky *et al.* 2012). A recent analysis of the genome of *C. littledalei* indicates that retrotransposons account for nearly twice the amount of DNA transposons (33% vs 18%; Global Carex Group 2015; Can *et al.* 2020), and so the higher fraction of DNA transposons observed in *C. cristatella* and *C. scoparia* may not be uniform across this genus. Among the DNA transposons in *C. cristatella* and *C. scoparia*, 3 families (CACTA, *hAT*, and MULE) represent the majority of the DNA TEs, while the remainder of the families examined (*PIF/Harbinger*, *Tc1/Mariner*, and *Helitron*) occupy less than 1% of the genome, suggesting the differential amplification of distinct DNA transposon families. In contrast to the *C. cristatella* and *C. scoparia* genomes, the majority of *J. effusus* and *J. inflexus* TEs are retrotransposons. In particular, both of the *Juncus* genomes consist of over 10% *Copia* elements, which is twice as much as that of *Gypsy* elements. The *C. cristatella* and *C. scoparia* genomes contain a lower percentage of retrotransposons, yet *Copia* elements are more abundant than *Gypsy* elements as well (Table 4, Supplementary Fig. 11). This is uncommon since the majority of plant genomes harbor more *Gypsy* elements than *Copia* elements (Cerbin and Jiang, unpublished). The high fraction of *Copia* elements in *C. cristatella*, *C. scoparia*, *J. effusus*, and *J. inflexus* resemble that in *M. acuminata*, another monocot plant (D’Hont *et al.* 2012; Belser *et al.* 2021). In general, DNA transposons are located in genic or euchromatic regions (Zhao *et al.* 2016). Among LTR retrotransposons, the distribution of *Copia* elements is often biased toward euchromatic chromosomal arms that are relatively close to genes, whereas *Gypsy* elements are more likely located in the gene-poor, heterochromatic or pericentromeric regions (Baucom *et al.* 2009; Bousios *et al.* 2012). The prevalence of DNA transposons in *C. cristatella* and *C. scoparia* and the larger fraction of *Copia* elements in these 4 *Carex* and *Juncus* genomes is likely a consequence of inbreeding, which is consistent with the very low degree of heterozygosity that is found in these species (Supplementary Fig. 1).

### Genome annotation

Gene prediction with the MAKER pipeline used Oxford Nanopore long-read transcript assemblies and protein alignment evidence to guide the gene prediction programs Augustus and SNAP. Between 25,422 and 26,500 genes were identified in the final high-confidence, non-TE-related, defused gene sets for these 4 species (Table 5), and these numbers of genes are comparable to the number of non-TE-related genes found in diploid plant species with small to moderate-sized plant genomes (The Arabidopsis Genome Initiative 2000; VanBuren *et al.* 2018; Can *et al.* 2020). Average gene size was slightly larger in *C. cristatella* and *C. scoparia* than in *J. effusus* and *J. inflexus*, and this was due to the larger introns in the *Carex* species compared to the *Juncus* species (Table 5). Average CDS length, number of exons per gene and number of introns per gene were similar between all 4 species.

**Table 5.** Characteristics of annotated genes from *Carex* and *Juncus*.

	*C. cristatella*	*C. scoparia*	*J. effusus*	*J. inflexus*
Number of genes	26,500	25,799	25,967	25,422
Average CDS length (bp)	1,054	1,069	1,018	1,023
Exon number per gene	5.27	5.43	5.43	5.53
Intron number per gene	4.27	4.43	4.43	4.53
Average intron length per gene	1,958	2,031	1,263	1,300
Average gene length (bp)	3,310	3,464	2,625	2,717

### Gene GC content

**Fig. 1.**
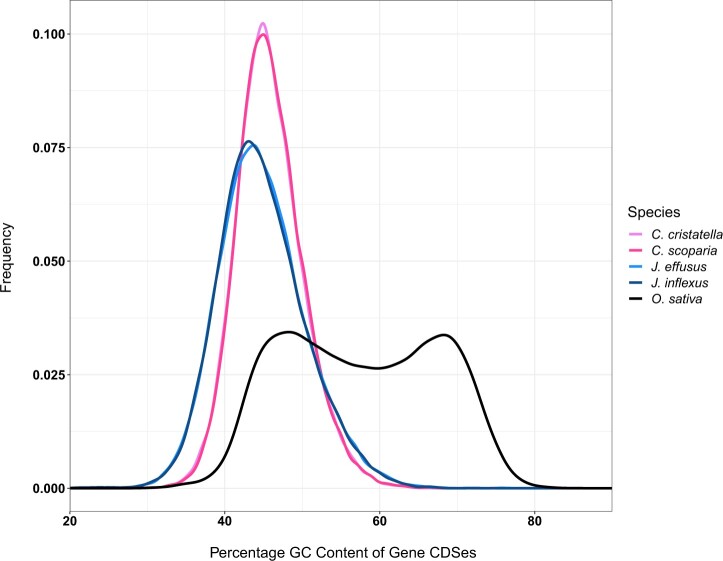
Distribution of full CDS GC content of *C. cristatella*, *C. scoparia*, *J. effusus*, *J. inflexus*, and *O. sativa*. Only primary isoforms and non-TEs-related genes were used in GC content calculations.

Ancestral monocot species are believed to have had a bimodal gene GC distribution, which is strongly preserved in Poaceae species, but many other monocot species have a unimodal gene GC distribution (Serres-Giardi *et al.* 2012; Clément *et al.* 2014; McKain *et al.* 2016). We examined gene GC and GC3 distribution for the *Carex* and *Juncus* species here and compared these distributions with those from *O. sativa* (Fig. 1, Supplementary Fig. 12). Compared to *O. sativa*, the *Carex* and *Juncus* species have unimodal GC and GC3 distributions, which is common for many non-Poaceae monocot species (Serres-Giardi *et al.* 2012; Clément *et al.* 2014; McKain *et al.* 2016). *Carex cristatella* and *C. scoparia* have nearly identical gene GC distributions with peaks at about 43%, and *J. effusus* and *J. inflexus* also have nearly identical GC distributions with peaks at 45%. Many cyperid genes have lower gene GC content than even the low-GC genes from *O. sativa*.

### Gene orthologs

**Fig. 2.**
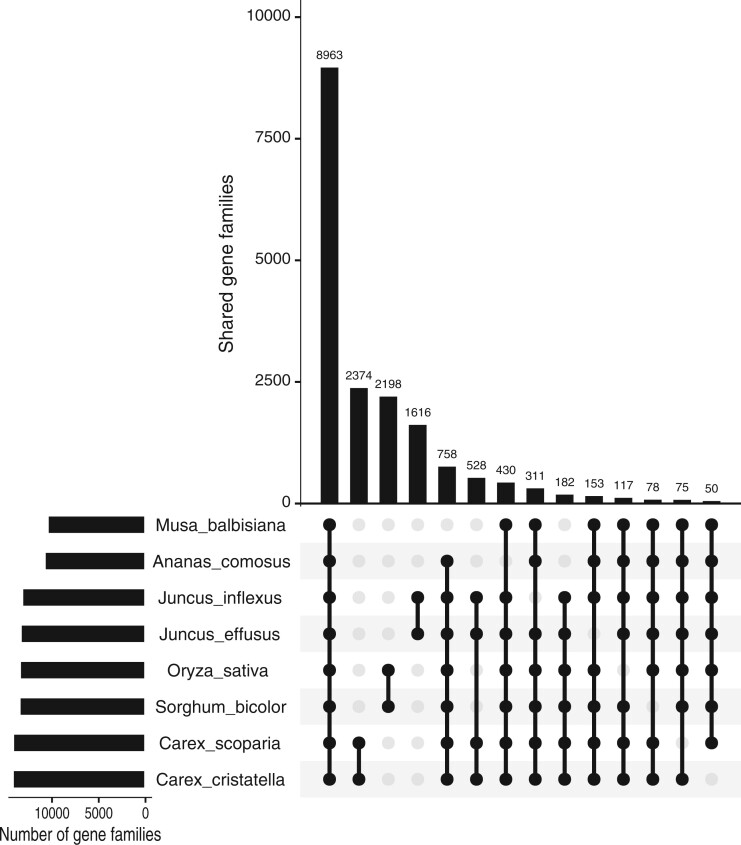
Numbers of shared single-copy and multiple-copy gene families among the 8 monocot genomes. Gene families were identified in *A. comosus*, *C. cristatella*, *C. scoparia*, *J. effusus*, *J. inflexus*, *M. balbisiana*, *O. sativa*, and *S. bicolor* using OrthoFinder.

Ortholog analysis was performed with proteins from 8 monocot species, *A. comosus*, *C. cristatella*, *C. scoparia*, *J. effusus*, *J. inflexus*, *M. balbisiana*, *O. sativa*, and *S. bicolor*. Nearly 9,000 orthologous groups were identified that contained genes from only the 8 monocot species, and these groups represent gene families that are generally conserved across the monocots (Fig. 2). *Carex*-, *Juncus*-, and Poaceae-specific orthologous gene families were identified with between 1,616 and 2,374 gene families each. GO enrichment analysis of the *Carex*-specific gene families showed an enrichment for metabolic processes, transcription factors, stress response genes, and diterpenoid biosynthesis (Supplementary Fig. 13). The *Juncus*-specific gene families have a significant abundance of GO terms for metabolic processes, defense responses, and homeostatic processes (Supplementary Fig. 14). GO terms related to various metabolic processes and ion transport were abundant within the Poaceae-specific gene families (Supplementary Fig. 15). Interestingly, the enrichment of gene families annotated with stress response terms within both *Carex* and *Juncus* but not in the Poaceae suggests that *Carex* and *Juncus* have developed additional mechanisms to respond to stress compared to the grasses.

### Monocot phylogeny

**Fig. 3.**
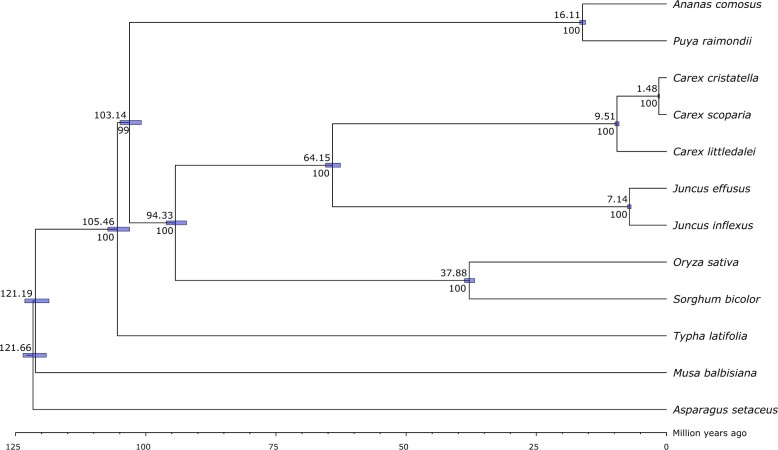
Phylogeny and estimation of divergence times of the sequenced *Carex* and *Juncus* genomes with *Asparagus setaceus* and 7 other Poales species based on 459 single-copy ortholog sequences. Numbers above and below the nodes are divergence times in millions of years and bootstrap values, respectively. Species divergence times estimated by MCMCTree at 95% confidence interval are shown as bars at each node of the tree.

A phylogenetic tree generated using orthologs identified from the predicted gene sets from *A. comosus*, *A. setaceus*, *C. cristatella*, *C. littledalei*, *C. scoparia*, *J. effusus*, *J. inflexus*, *M. balbisiana*, *O. sativa*, *P. raimondii*, *S. bicolor*, and *T. latifolia* was created (Fig. 3). This phylogenetic tree is consistent with Poales phylogenies from a number of other reports that indicate Cyperaceae and Juncaceae as being the most recently diverged families and an earlier time of divergence between Bromeliaceae and Poaceae, Cyperaceae, and Juncaceae (Bremer 2002; Eguchi and Tamura 2016). Estimated times of divergence in this phylogeny are consistent with those of Eguchi and Tamura (2016) and Can *et al.* (2020), but other reports found generally earlier branching dates than those calculated here (Janssen and Bremer 2004; Givnish *et al.* 2018).

### tRNA identification and analysis

**Fig. 4.**
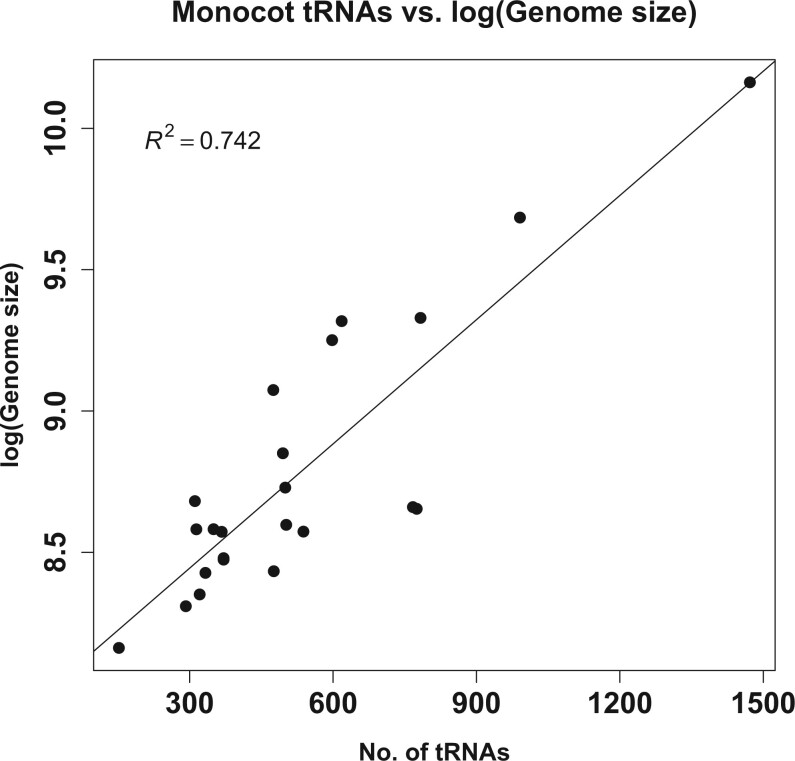
Correlation between genome size and total number of predicted tRNA genes in 23 monocot genomes.

**Fig. 5.**
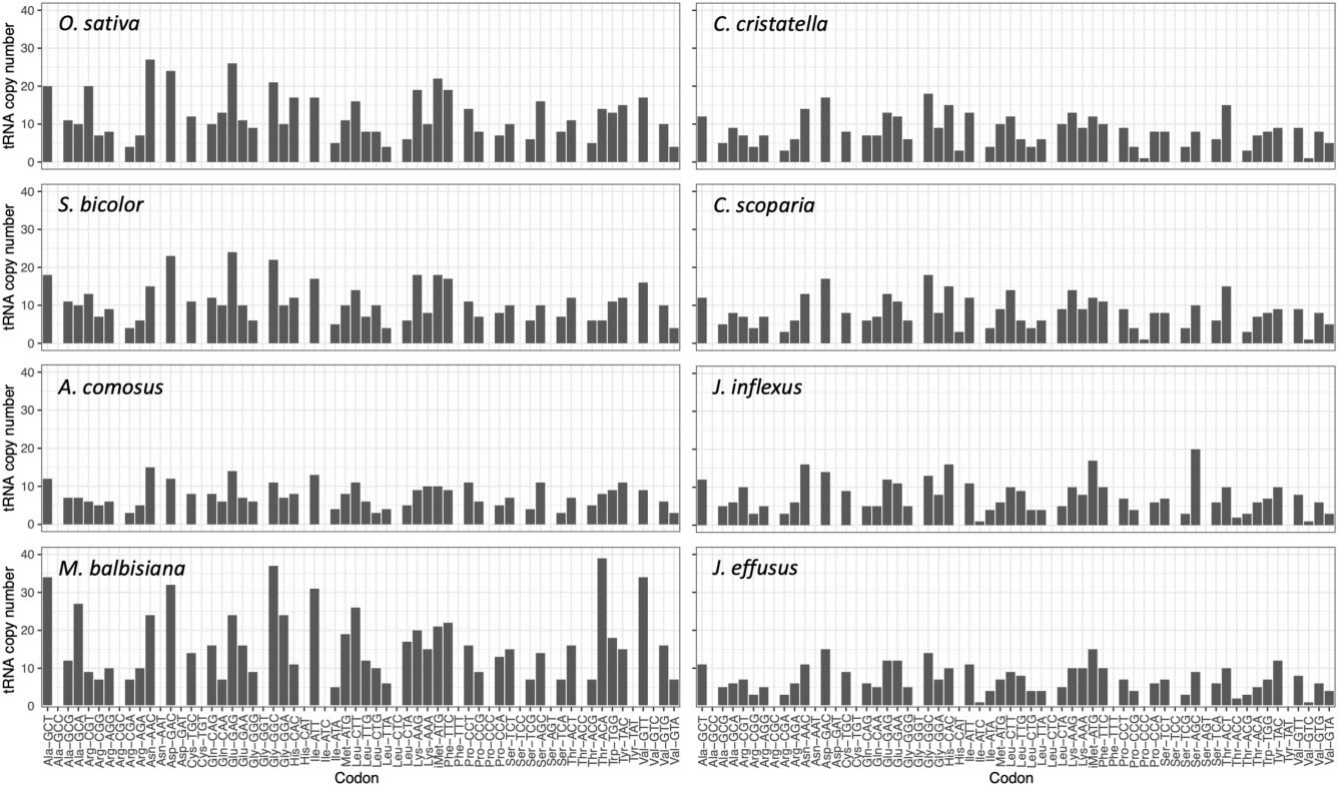
Summary of the predicted tRNA genes grouped by codons and amino acid specificity from the genomes of *A. comosus*, *C. cristatella*, *C. scoparia*, *J. effusus*, *J. inflexus*, *M. balbisiana*, *O. sativa*, and *S. bicolor*.

For purposes of comparison, we identified tRNA coding genes with tRNAscan-SE and Infernal in *C. cristatella*, *C. scoparia*, *J. effusus*, and *J. inflexus* as well as *A. comosus*, *M. balbisiana*, *O. sativa*, and *S. bicolor*. The total number of tRNA genes identified ranged from 321 to 767 (Supplementary Table 5), and with the exception of *M. balbisiana*, the numbers of predicted tRNA genes found in these monocot genomes are correlated with genome size as has been observed previously (Fig. 4; Michaud *et al.* 2011). We categorized high-confidence tRNA gene predictions by amino acid as well as by anticodon, and tRNA genes with identical sequences were also grouped into unique, nonredundant tRNA sets (Supplementary Table 5). The relative numbers of tRNA genes for individual amino acids were found to be similar across species (Fig. 5, Supplementary Table 5). For example, tRNA genes for cysteine and tryptophan were typically much less abundant than most other tRNAs in the species examined here, and tRNA genes for glycine and leucine were generally more abundant than most other tRNAs. We also found that the relative ratios of tRNA isoacceptors for each amino acid are very similar across all 8 monocots analyzed (Fig. 5, Supplementary Table 5). A Spearman's ranked correlation analysis indicated very significant correlation of the numbers of each tRNA isoacceptor in *C. cristatella*, *C. scoparia*, *J. effusus*, *J. inflexus*, *S. bicolor*, *A. comosus*, and *M. balbisiana* relative to the isoacceptors in *O. sativa* (Supplementary Table 6). This suggests that the relative ratios of tRNA genes have been under selection across these species to maintain a balance of tRNA genes at both the isoacceptor and amino acid levels.

There were 13 isoacceptors that were not represented among any of these 8 species (Fig. 5, Supplementary Table 5). *Carex cristatella* and *C. scoparia* had tRNA^His^(ATG) and tRNA^Pro^(GGG) genes that were not found in the other species. *Juncus effusus* and *J. inflexus* contained tRNA^Ile^(GAT) and tRNA^Thr^(GGT) genes that were missing from the other monocots analyzed here. The *Carex* and *Juncus* species all contained a single tRNA^Val^(GAC) gene that was absent from the *A. comosus*, *M. balbisiana*, *O. sativa*, and *S. bicolor* genomes. This indicates despite the conservation of the majority of tRNA genes in monocots, there is a certain degree of presence/absence variation of individual tRNA genes across species.

### Satellite repeat analysis

**Fig. 6.**
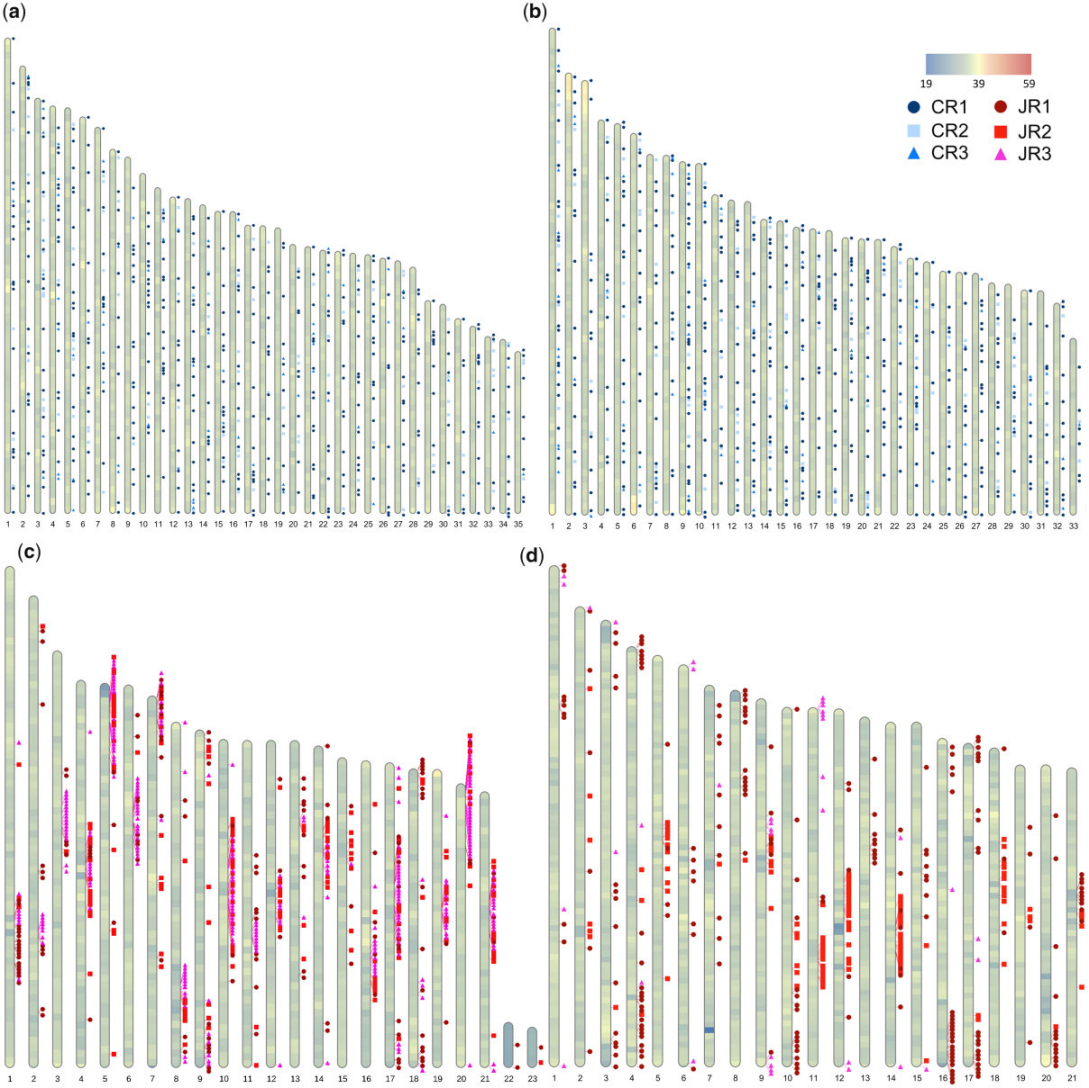
Genome-wide characterization of GC content and satellite family distribution in (a) *C. cristatella*, (b) *C. scoparia*, (c) *J. effusus*, and (d) *J. inflexus*. Coloring along the scaffold diagrams indicates GC percentage, and colors range from 19% to 59 % GC content in 200-kb window size as depicted in the legend. Locations of *Carex*-specific repeat families, CR1, CR2, and CR3, are displayed along scaffold diagrams for (a) and (b). Locations of *Juncus*-specific repeat families, JR1, JR2, and JR3, are displayed along scaffold diagrams for (c) and (d).

Within each species, the 3 largest satellite repeat families, as measured by total length, were identified by clustering similar repeat sequences recognized by TRF (Supplementary Table 7). These large satellite repeat families composed 3.2–9.5% of the genomes from these 4 species (Table 4). In *C. cristatella* and *C. scoparia*, the 3 largest satellite repeat families had nearly identical consensus sequences (Supplementary Table 7). The *Carex* satellite repeat families (CR1, CR2, and CR3) have remarkably similar numbers of repeat instances, average size, and total length within *C. cristatella* and *C. scoparia*. These *Carex* repeat families have period lengths of 31, 29, and 30 bases, respectively, although higher-order period lengths were recognized by TRF for each of these *Carex* repeats (Supplementary Files 1–6). The repeat CR1 in particular had higher-order repeats of 61, 93, 124, and 248 bases. Mapping of the CR1, CR2, and CR3 repeats shows that instances of all of these repeats are generally spread randomly across all chromosomes. There are few instances of these repeats clustering in the same chromosomal regions (Fig. 6). Similarly, the 3 largest satellite repeat families in *J. effusus* and *J. inflexus* were also nearly identical between the 2 species (Supplementary Table 7). The *Juncus* satellite repeat families (JR1, JR2, and JR3) had predominant periods of 155, 181, and 364 bases, respectively, but higher- and lower-order repeats were also found within these families (Supplementary Files 7–12). The *Juncus* repeats were largely clustered in a single location on each chromosome, but there were also sporadic instances of repeats from each family throughout the *Juncus* genomes (Fig. 6). Clusters of instances of the JR1 and JR2 repeats are similarly frequent across the *J. inflexus* genome, but the JR3 repeats are infrequently found near JR1 and JR2 repeats in *J. inflexus*. This is in stark contrast with the distribution of these repeats in *J. effusus* where clusters containing 2 or 3 of these repeat families are very common.

Centromeres have been defined as the sequence bound by CenH3 during mitosis or meiosis (Zhang *et al.* 2013; Marques *et al.* 2016), and centromeres are often found within arrays of satellite repeats (Dong *et al.* 1998; Gong *et al.* 2012; Heckmann *et al.* 2013). The basic period lengths of centromere-associated repeats are assumed to wrap around a single nucleosome (Henikoff *et al.* 2001), but centromere-associated repeats are recognized that have widely different monomeric lengths (Gong *et al.* 2012). While a single satellite repeat family is often recognized from centromere regions of many species (Jiang *et al.* 2003), multiple families of centromere-associated repeats within a single genome are known (Neumann *et al.* 2012; Gong *et al.* 2012; Yang *et al.* 2018). The CR1, CR2, and CR3 repeats from *C. cristatella* and *C. scoparia* are not clustered, but the numbers and total lengths of these repeat families are very similar between the 2 *Carex* species. These characteristics suggest conserved roles for these repeats in these 2 species and point to the possibility that these repeats are also centromere-associated. While holocentrism has been observed in *Carex* but not in *C. cristatella* and *C. scoparia* specifically (Heilborn 2010; Marques *et al.* 2015; Escudero *et al.* 2016; Ribeiro *et al.* 2017), the distributions of these repeats would be consistent for centromere-associated repeats in holocentric species. Nonetheless, sequencing of DNA bound to CenH3 histones will be required to definitively resolve whether any of these satellite repeat classes are associated with centromeres in *C. cristatella* and *C. scoparia*. All examined members of *Rhynchospora* from Cyperaceae exhibit holocentrism (Marques *et al.* 2015; Ribeiro *et al.* 2017). The Tyba centromere associated repeat was isolated by immunoprecipitation of CenH3 from several species of *Rhynchospora* (Marques *et al.* 2015). However, the Tyba repeat does not have sequence similarity with the CR1, CR2, and CR3 repeats from *Carex*, nor does it have similarity to any other tandem repeat families that had been identified in *Carex* (data not shown).

The features of satellite repeats JR1, JR2, and JR3 from *J. effusus* and *J. inflexus* resemble those associated with classical regional centromeres. The similar sequences, abundances, and distributions of the JR1, JR2, and JR3 repeats in *J. effusus* and *J. inflexus* suggest a conserved function of these satellite repeats in these 2 species. More importantly, the single large cluster of these satellite sequences on each chromosome suggests that they are associated with centromeres and supports the conclusion by Guerra *et al.* (2019) that *Juncus* species have monocentric chromosomes. Centromere-associated sequences have also been identified from several species of *Luzula* from Juncaceae (Haizel *et al.* 2005). The LCS1 satellite repeat from *Luzula* colocates with CENH3 in *Luzula* by fluorescent *in situ* hybridization, but unlike the JR1, JR2, and JR3 repeats in *Juncus*, the LCS1 repeat is found in at least 5 regions per holocentric chromosome in *Luzula* (Haizel *et al.* 2005). The JR1, JR2, and JR3 satellite repeats as well as other less abundant tandem repeats from *Juncus* do not align with the LCS1 repeat from *Luzula* (data not shown). Sequencing of CenH3 immunoprecipitated DNA from *J. effusus* and *J. inflexus* will also be required to definitively determine whether the JR1, JR2, and JR3 satellite repeats are centromere-associated.

## Supplementary Material

jkac211_Supplemental_File_S1

jkac211_Supplemental_File_S2

jkac211_Supplemental_File_S3

jkac211_Supplemental_File_S4

jkac211_Supplemental_File_S5

jkac211_Supplemental_File_S6

jkac211_Supplemental_File_S7

jkac211_Supplemental_File_S8

jkac211_Supplemental_File_S9

jkac211_Supplemental_File_S10

jkac211_Supplemental_File_S11

jkac211_Supplemental_File_S12

jkac211_Supplemental_Tables

## Data Availability

The data supporting the observations and conclusions described in this report can be found within the manuscript, in accompanying supplemental data at *G3* online and in public repositories. Sequence data and final genome assemblies underlying this article are available at GenBank under BioProject PRJNA723756. These Whole Genome Shotgun projects have been deposited at GenBank under the accession JAJHPF000000000, JAJHPE000000000, JAJHPD000000000, and JAJHPC000000000. The genome assembly versions described in this paper are versions JAJHPF010000000, JAJHPE010000000, JAJHPD010000000, and JAJHPC010000000. Genome annotation files, predicted transcript and protein fasta files, and functional annotation files underlying this article are available in the Dryad Digital Repository at https://doi.org/10.5061/dryad.msbcc2g0z.
